# Hydroxyl Group as the ‘Bridge’ to Enhance the Single-Molecule Conductance by Hyperconjugation

**DOI:** 10.3390/molecules29112440

**Published:** 2024-05-22

**Authors:** Xin Lv, Chang Li, Meng-Meng Guo, Wenjing Hong, Li-Chuan Chen, Qian-Chong Zhang, Zhong-Ning Chen

**Affiliations:** 1 Key Laboratory of Structural Chemistry, Fujian Institute of Research on the Structure of Matter, Chinese Academy of Sciences, Fuzhou 350002, China; lvxin@fjirsm.ac.cn (X.L.); lichang@fjirsm.ac.cn (C.L.); mengmengguo@fjirsm.ac.cn (M.-M.G.); czn@fjirsm.ac.cn (Z.-N.C.); 2 College of Chemistry and Materials Science, Fujian Normal University, Fuzhou 350007, China; 3 Fujian College, University of Chinese Academy of Sciences, Fuzhou 350002, China; 4 University of Chinese Academy of Sciences, Beijing 100049, China; 5 State Key Laboratory of Physical Chemistry of Solid Surfaces, College of Chemistry and Chemical Engineering, Xiamen University, Xiamen 361005, China; whong@xmu.edu.cn; 6 Fujian Science & Technology Innovation Laboratory for Optoelectronic Information of China, Fuzhou 350108, China

**Keywords:** single-molecule conductance, conductance enhancement, hyperconjugation, hydroxyl

## Abstract

For designing single-molecule devices that have both conjugation systems and structural flexibility, a hyperconjugated molecule with a σ–π bond interaction is considered an ideal candidate. In the investigation of conductance at the single-molecule level, since few hyperconjugation systems have been involved, the strategy of building hyperconjugation systems and the mechanism of electron transport within this system remain unexplored. Based on the skipped-conjugated structure, we present a rational approach to construct a hyperconjugation molecule using a hydroxyl group, which serves as a bridge to interact with the conjugated fragments. The measurement of single-molecule conductance reveals a two-fold conductance enhancement of the hyperconjugation system having the ‘bridging’ hydroxyl group compared to hydroxyl-free derivatives. Theoretical studies demonstrate that the hydroxyl group in the hyperconjugation system connects the LUMO of the two conjugated fragments and opens a through-space channel for electron transport to enhance the conductance.

## 1. Introduction

Aiming at the miniaturization of electric devices, the development of molecular electronics depends on fabricating molecular devices with highly conductive molecules [1,2,3,4]. Since the π orbital has the energetic advantages in electron transport, the strategies to design molecules performing high conductance are usually based on the fully conjugated structures, including the introduction of constructive quantum interference [5,6,7], the construction of self-gating systems [8,9,10] and the ‘topological insulator’ built by the radical system [11,12]. However, despite the good performance in electric properties, the conjugated structures which bring in high structural rigidity prevent the conformational transition that offers the opportunities to modulate the conductance through folded structures, for instance, intramolecular π–π stacking [13,14,15]. Thus, exploring a strategy to design molecules having the feature of conjugation and structural flexibility synchronously is of great significance.

Hyperconjugation, which refers to the interaction between σ- and π-orbital, introduces the delocalized molecular orbitals in non-conjugated structures [16,17,18,19,20]. Although it is a promising candidate for combining structural flexibility with conjugated backbones in designing single-molecule devices, the molecular structures containing a hyperconjugation system are rarely investigated [21,22]. In particular, the mechanism of electron transport in a hyperconjugation system is still unclear. Therefore, designing a molecule with π–σ hyperconjugation to explore the mechanism of electron transport in this system is of great importance in developing high-performance single-molecule devices.

Here we report a hyperconjugation system based on a skipped-conjugation structure [23] with a non-conjugated hydroxyl group as a ‘bridge’ to connect the conjugated fragments. The distinct enhancement of conductance for the hydroxyl-contained hyperconjugation systems was confirmed by comparison with that for the corresponding hydroxyl-free derivatives. Theoretical studies demonstrated that hyperconjugation, in which the anti-bonding orbital (σ*) of the C-O (hydroxyl group) bond interacts with the anti-bonding orbital (π*) of the adjacent conjugated structure, builds the interaction between the hydroxyl group and the two conjugated fragments and connects the two fragments, thus opening a new through-space channel for the transmission of electrons to enhance the conductance.

## 2. Results

**Figure 1:**
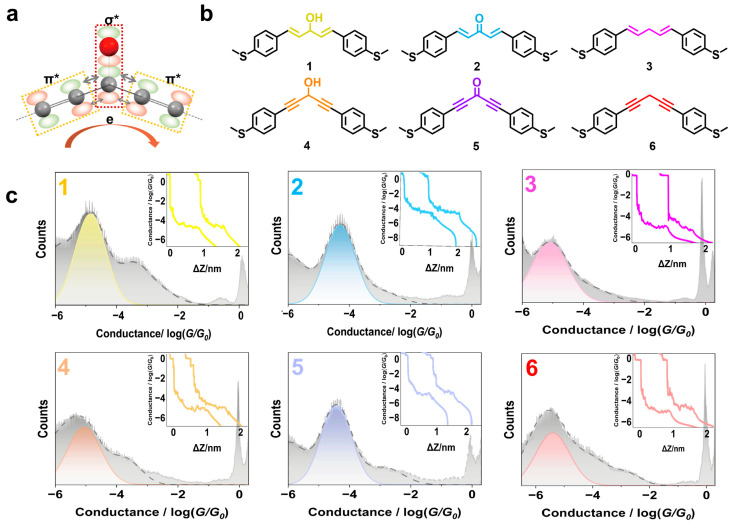
(**a**) Diagram of the interaction between the σ* and π* in the hydroxyl-bridged structure; (**b**) structures of molecules **1**–**6**; (**c**) one-dimensional (1D) histograms of the measured conductance (grey) compiled by 4180, 3830, 3664, 2171, 2035 and 2406 conductance-displacement traces for molecules **1**–**6**, respectively. The dashed lines are the fitting curves and the colored peaks are the fitting peaks of the most probable conductance for each molecule, with insets showing the statistical junction length (Supplementary Material Figures S14 and S15).

To clarify the role of hyperconjugation playing in single-molecule conductance, we elaborately designed two groups of directly comparable molecules in this work, as depicted in Figure 1. Molecule **1**, containing one hydroxyl group as the ‘bridge’ to link the two conjugated fragments (phenylethenyl groups), was designed and synthesized according to the reported methods [24,25,26,27]. As a structural reference, the hydroxyl group was replaced by the carbonyl group to link the two conjugated fragments in molecule **2** [28]. Upon the removal of the hydroxyl group, we obtained molecule **3** with methylene to space the conjugated fragments. Molecules **4**–**6** were designed as the structural derivatives of molecules **1**–**3** with the ethenyl groups in the former replaced by the ethynyl groups in the latters, respectively. The single-molecule conductance was measured using the scanning tunneling microscopy break junction (STM-BJ) technique in the 1,2,4-Trichlorobenzene (TCB) solution containing 0.1 mM of the prepared molecules.

As shown in Figure 1, the most probable conductance of molecule **1** is 10^−4.85^ *G*_0_ (1.09 nS, *G*_0_ represents the quantum conductance and equals 77,500 nS) [23], which is lower than the conductance of molecule **2** (10^−4.29^ *G*_0_, 3.97 nS) and higher than the conductance of molecule **3** (10^−5.10^ *G*_0_, 0.62 nS). This implies that, compared to the fully conjugated molecule **2**, breaking the conjugated system with a saturated carbon atom at the central site of molecule **1** suppresses the conductance effectively. However, compared with molecule **3**, the existence of the hydroxyl group enhances the conductance as the conductance of molecule **1** is about two times as high as that of molecule **3** [29]. This conductance modulation is observed not only in ethenyl-connected molecules **1**–**3**, but also characterized in ethynyl-connected molecules **4**–**6**. The conductance of hydroxyl group-bridged molecule **4** (10^−5.05^ G_0_, 0.69 nS) is also lower than that of fully conjugated molecule **5** (10^−4.42^ G_0_, 2.95 nS), but higher than that of hydroxyl-free molecule **6** (10^−5.40^ G_0_, 0.31 nS).

To find the origin of the conductance enhancement by the hydroxyl group in skipped-conjugated structures, the junction length was investigated because the conductance is very sensitive to a change in the junction length. Since the conductive backbone of molecules **1**–**6** are almost the same, the variation of the statistical junction lengths of molecules **1**–**6** is relatively small, and are 1.04, 0.87, 1.03, 0.90, 1.02 and 0.88 nm, respectively. The measured junction lengths (1.54, 1.37, 1.53, 1.40, 1.52 and 1.38 nm for molecules **1**–**6**, Figure S17) match well with the simulated S-S distances (1.51, 1.59, 1.40, 1.43, 1.48 and 1.46 nm for molecules **1**–**6**) after correcting the junction length using a 0.5 nm ‘snap-back’ length [30]. With such as small length fluctuation, the contribution of the junction length is negligible in the conductance enhancement by the hydroxyl group.

**Figure 2:**
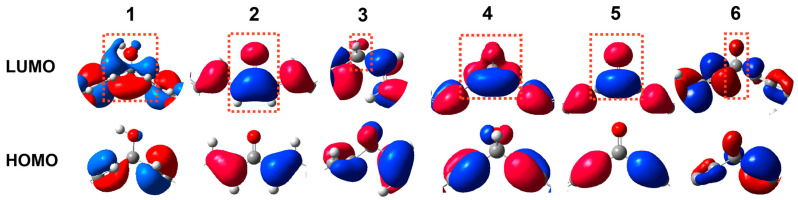
Electronic structure of the central parts of the HOMOs and LUMOs for molecules **1**–**6** with isovalues of 0.05 for molecules **1**–**6** (Supplementary Material Figure S16).

Molecular orbitals, especially the frontier molecular orbitals (FMOs) such as the lowest unoccupied molecular orbital (LUMO) and highest occupied molecular orbital (HOMO) are crucial to the conductance magnitude [29]. To investigate the influence of the hydroxyl group on the FMOs, the theoretical studies were conducted using density functional theory (DFT). The geometry optimization and orbital calculations of molecules **1**–**5** are performed by the Gaussian16 package [31], while the theoretical study of molecule **6** was reported in the previous research [29]. The electronic structure of the FMOs, which is highly correlated to the conductive property, was analyzed by simulating the distribution of HOMO and LUMO. In molecules **3** and **6**, the reported study shows an imbalanced distribution in both HOMO and LUMO (Figure S16) [32]. In particular, the central carbon atom, which skips the conjugated system, plays the role of a node to divide the structure into two conjugated fragments with no orbital distributed on it. Despite there being still no orbital distribution on this atom in HOMO, the orbital distribution of the two conjugated fragments in molecules **1**–**2** and **4**–**5** is connected through it in LUMO when a hydroxyl group is substituted. This results in orbital distribution in the skipped-conjugated molecules **1** and **4** is visually the same as that of fully conjugated molecules **2** and **5**, respectively (Figure 2).

**Figure 3:**
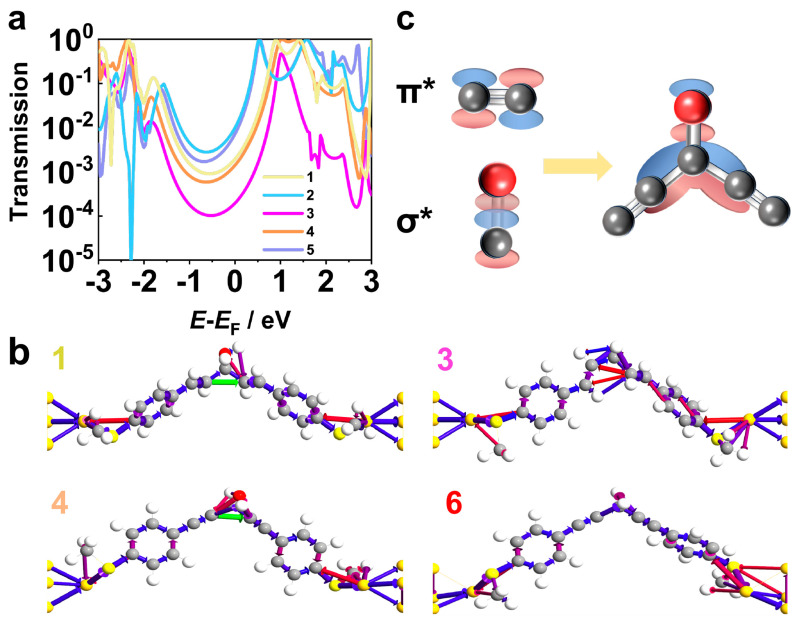
(**a**) Calculated transmission coefficients of molecules **1**–**5**. (**b**) Transmission pathways (blue arrows: forward transmission from source to drain, red arrows: reverse transmission) of molecules **1**, **3**, **4** and **6** with the green arrows demonstrating the through–space transmission channel opened by the hyperconjugation (the scaling magnitude for the transmission threshold is 0.05) (Transmission pathways of molecules **2** and **5** in Supplementary Material Figure S19). (**c**) Demonstration of the hyperconjugation in which the anti–bonding orbital (σ*) of C–O bond interacts with the anti–bonding orbital (π*) of C–C bond.

The transmission coefficients (*T*(*E*)) and the transmission pathway for the electron, which investigated the relationship between the electronic structure and the conductance, were calculated by combining density functional theory (DFT) with non-equilibrium Green’s functions (NEGF), where the Green’s function estimates the behavior of the electron wave in a single molecule and yields the *T*(*E*) [12,33,34,35]. The molecular junctions of **1**–**5** were simulated while the junction of molecule **6** was reported in previous work [29]. As shown in Figure 3, the transmission coefficients, which closely correspond with the single-molecule conductance, at a Fermi energy of (*E* − *E*_F_ = 0 eV), decrease following the sequence of **2** > **5** > **1** > **4** > **3**, which matches well with the sequence of the measured single-molecule conductance. More importantly, in the curves of the transmission coefficient for these molecules, the peaks representing LUMO are closer to *E*_F_ than the peaks of HOMO. This indicates that, for the corresponding molecules, the conductance is dominated by LUMO, which is also found in molecule **6** in the previous study. Hence, since the hydroxyl group connects the two conjugated fragments forming a delocalized system, the LUMO provides a continuous channel for the electron to transport from the source to the drain, which is explicitly demonstrated in the transmission pathway [36]. As shown in Figure 3b, a broad arrow between the two carbon atoms connecting the central carbon atom, which indicates the strong through-space transmission of electrons between the end atom of the two conjugated fragments, is observed in molecules **1**, **2**, **4** and **5**, while no such a through-space transmission is characterized in molecules **3** and **6**. The strong through-space transmission reveals that the connection of the two conjugated fragments in LUMO opens an extra transmission channel for the electron, enhancing the conductance.

Finally, the hyperconjugation effect was employed to elucidate the origin of the enhanced conductance facilitated by the hydroxyl group connecting the two conjugated systems [17,18,37]. Hyperconjugation refers to the interaction between the σ– and π–orbital [19]. In molecules **1** and **4**, the simulated electronic structures demonstrate the formation of the extended LUMO. The unsaturated C–C bonds (ethenyl or ethynyl group) offer the anti–bonding orbital (π*) and the C–O (hydroxyl group) bond also provides the anti−bonding orbital (σ*). In molecular structure, as the unsaturated C–C bonds connect to the carbon atom of the C–O bond, the matched phase of the π* and σ* results in parts of the π* orbital hybridizing with parts of the σ* orbital, which eventually extended the conjugation system in the skipped–conjugated structure.

## 3. Materials and Methods

### 3.1. General

Physical Measurement. The ^1^H and ^13^C NMR spectra were recorded on a Bruker AVANCE ΙΙΙ 400 M NMR or ECZ 600R NMR spectrometer with SiMe_4_ as the internal reference. All coupling constants are absolute values and the *J* values are expressed in hertz (Hz). The description of the signals includes s = singlet, d = doublet, m = multiplet and dd = doublet of doublets.

General Procedures and Reagents. All the manipulations were carried out using Schlenk techniques and vacuum–line systems under a dry argon atmosphere unless otherwise specified. All reagents were of commercial origin and were used as received. The triethylamine (TEA) was distilled over CaH_2_. Silica gel (100–200 mesh) was used for the column chromatography.

#### Synthesis and Characterizations


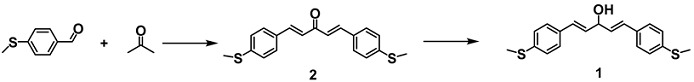


(1E,4E)-1,5-bis(4-(methylthio)phenyl)penta-1,4-dien-3-one (**2**) 

General procedure for cross-aldol condensation of ketones with aldehydes: To a stirred mixture of the 4-(methylthio)benzaldehyde (1.73 mL, 13 mmol) and anhydrous ethanol (1.46 mL, 25 mmol), thionyl chloride (0.36 mL, 5 mmol) and acetone (0.74 mL, 10 mol) were dropped synchronously. The flask was cooled below 0 °C (ice–salt cold bath). The solution turned yellow immediately. When stirred for 3–10 min, the mixture coagulated. Then the flask was placed in an ice–salt cold bath for 2 h. After completion of the reaction, saturated aqueous Na_2_CO_3_ was added and the mixture was filtered. The solid was washed twice successively with water (30 mL), anhydrous ethanol (20 mL) and ethyl ether (10 mL). The crude product was purified by flash chromatography to obtain compound **2** (yellow solid, 3.475 g, 81.9%) [28]. ^1^H NMR (400 MHz, CDCl_3_) δ 7.69 (d, *J* = 15.9 Hz, 2H), 7.53 (d, *J* = 8.4 Hz, 4H), 7.25 (d, *J* = 7.8 Hz, 4H), 7.03 (d, *J* = 15.9 Hz, 2H), 2.52 (s, 6H). ^13^C NMR (100 MHz, CDCl_3_) δ 189.71(1C), 188.71(2C), 142.64(2C), 142.30(2C), 131.30(2C), 128.76(4C), 125.96(4C), 124.53(2C), 14.68(2C).

(1E,4E)-1,5-bis(4-(methylthio)phenyl)penta-1,4-dien-3-ol (**1**)

To a cooled (0 °C) slurry of (1E,4E)-1,5-bis(4-(methylthio)phenyl)penta-1,4-dien-3-one (1.632 g, 5 mmol) in methanol (10 mL), NaBH_4_ (0.189 g, 5 mmol) was added in two portions. After 2 h, the resulting solution was poured into 1 M NaOH (50 mL) and the crystalline product was collected and recrystallized from heptane (50 mL) to obtain compound **1** (white solid, 1.412 g, 86%) [27]. ^1^H NMR (400 MHz, CDCl_3_) δ 7.32 (d, *J* = 8.4 Hz, 4H), 7.20 (d, *J* = 8.4 Hz, 4H), 6.61 (d, *J* = 15.9 Hz, 2H), 6.26 (dd, *J* = 15.9, 6.5 Hz, 2H), 5.01–4.93 (m, 1H), 2.48 (s, 6H). ^13^C NMR (100 MHz, CDCl_3_) δ 138.25(2C), 133.48(2C), 130.43(2C), 129.73(4C), 127.08(4C), 126.60(2C), 73.39(1C), 14.33(2C).


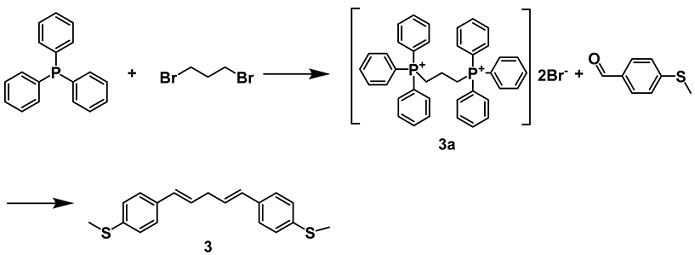


1,3-bis(triphenylphosphino)propane dibromide (**3a**)

A mixture of the triphenylphosphine (6.557 g, 25 mmol) and 1,3-dibromopropane (2.019 g, 10 mmol) in DMF (30 mL) was stirred and refluxed for 4 h. After reaction was complete, the reaction mixture was cooled and the crude white precipitate was filtered and washed with DMF (3 × 10 mL). The white powder was dried under stream of air and recrystallized in water as cubic shape crystals **3a** (white solid, 6.901 g, 95%) [38].

1,5-bis(4-(methylthio)phenyl)penta-1,4-diyne (**3**)

Compound **3a** (1.831 g, 2.52 mmol) was added to 50 mL of anhydrous THF under nitrogen. The contents were cooled to −78 °C with an ice bath and *n*-BuLi was added dropwise (1.6 M in hexanes, 5.04 mmol). After stirring at this temperature for 1 h, the ice bath was removed and it slowly warmed to room temperature. After 15 min at room temperature, 4-(methylthio)benzaldehyde (0.355 g, 2.33 mol) was added in 10 mL of anhydrous THF dropwise. A reflux condenser was added and the system was heated to 80 °C. It was heated for 1.5 h and then cooled and quenched with 50 mL of water. The aqueous was extracted with diethyl ether (3 × 100 mL) and organic layer was washed with water (2 × 150 mL) and brine (1 × 100 mL) and dried over MgSO4. The solvents were removed under vacuum yielded a crude orange oil that slowly solidified. The crude product was purified by flash chromatography to obtain compound **3** (white solid,0.201 g, 28%) [39]. ^1^H NMR (600 MHz, CDCl_3_) δ 7.29 (d, *J* = 8.5 Hz, 2H), 7.22 (d, *J* = 8.3 Hz, 2H), 7.19 (d, *J* = 8.5 Hz, 2H), 7.14 (d, *J* = 8.2 Hz, 2H), 6.73 (dd, *J* = 15.7, 10.5 Hz, 1H), 6.43 (d, *J* = 15.6 Hz, 1H), 6.23 (dd, *J* = 15.1, 10.5 Hz, 1H), 5.92 (dt, *J* = 14.4, 6.9 Hz, 1H), 3.44 (d, *J* = 7.0 Hz, 2H), 2.48 (d, *J* = 1.9 Hz, 6H). ^13^C NMR (150 MHz, CDCl_3_) δ 137.48(1C), 137.26(1C), 135.92(1C), 134.52(1C), 133.44(1C), 131.86(1C), 130.55(1C), 129.29(2C), 128.43(2C), 127.27(2C), 126.77(2C), 126.71, 38.72(1C), 16.35(1C), 15.92(1C).


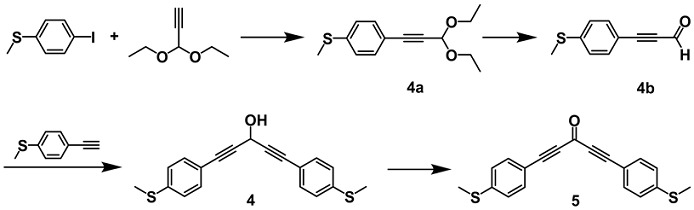


(4-(3,3-diethoxyprop-1-yn-1-yl)phenyl)(methyl)sulfane (**4a**)

A mixture of 1-iodo-4-methylsulfanylbenzene (3.751 g, 15.00 mmol), 3,3-diethoxyprop-1-yne (3.01 mL, 21.00 mmol), triethylamine (9 mL) and Pd(PPh_3_)_2_Cl_2_ (1.052 g, 10 mol%), CuI (0.286 g, 10 mol%) in 45 mL of THF was stirred in nitrogen at room temperature for 5 h. The solvent was removed in vacuo. The residue was extracted with water and dichloromethane, followed by the organic phase being washed with saturated brine, dried over anhydrous sodium sulfate and concentrated under reduced pressure. The crude product was purified by flash chromatography to obtain compound **4a** (yellow oily liquid, 3.470 g, 92.4%). ^1^H NMR (400 MHz, CDCl_3_): δ 7.36 (d, *J* = 8.5 Hz, 2H), 7.14 (d, *J* = 8.5 Hz, 2H), 5.47 (s, 1H), 3.86–3.72 (m, 2H), 3.65 (m, 2H), 2.45 (s, 3H), 1.26 (t, 6H). ^13^C NMR (100 MHz, CDCl_3_): δ 140.20, 132.28(2C), 125.65(2C), 118.07, 91.91, 85.10, 84.47, 61.01(2C), 15.23(3C).

3-(4-(methylthio)phenyl)propiolaldehyde (**4b**)

Compound **4a** (3.470 g, 13.86 mmol) was dissolved in a mixed solvent (60 mL) of acetone and water (v:v = 1:1), then 5% HCl (45 mL) was added to the solution and the mixture was allowed to stand overnight at room temperature. Upon completion of the reaction, acetone was removed in vacuum. The residue was extracted with water and dichloromethane, washed with saturated brine, dried over anhydrous sodium sulfate and concentrated under reduced pressure. The crude product was purified by flash chromatography to obtain compound **4b** (yellow solid, 2.390 g, 97.8%). ^1^H NMR (400 MHz, CDCl_3_): δ 9.40 (s, 1H), 7.50 (d, *J* = 8.6 Hz, 2H), 7.22 (d, *J* = 8.6 Hz, 2H), 2.51 (s, 3H). ^13^C NMR (100 MHz, CDCl_3_): δ 176.83, 144.37, 133.69(2C), 125.46(2C), 115.05, 95.81, 89.16, 14.90.

1,5-bis(4-(methylthio)phenyl)penta-1,4-diyn-3-ol (**4**)

To a solution of the 4-ethynylthioanisole (0.815 g, 5.50 mmol) in anhydrous THF (20 mL), n-BuLi (1.6 M, 3.44 mL, 5.50 mmol) was added slowly at −78 °C. The resulting mixture was stirred at −78 °C for 1 h, then compound **4b** (0.872 g, 4.95 mmol) was added and the reaction temperature was raised to room temperature until completion of the reaction. The resulting mixture was quenched with a saturated solution of NH_4_Cl and extracted with ethyl acetate. The combined organic layers were washed with brine, dried over anhydrous Na_2_SO_4_ and concentrated under reduced pressure. The crude product was purified by flash chromatography to obtain compound **4** (yellow solid, 624 mg, 35.0%) [24,25]. ^1^H NMR (400 MHz, CDCl_3_): δ 7.39 (d, *J* = 8.7 Hz, 4H), 7.17 (d, *J* = 8.5 Hz, 4H), 5.57 (s, 1H), 2.48 (s, 6H). ^13^C NMR (100 MHz, CDCl_3_): δ 140.32(2C), 132.26(4C), 125.71(4C), 118.13(2C), 86.04(2C), 84.54(2C), 53.41, 15.31(2C). HR-MS (ESI): calcd. for C_19_H_16_OS_2_: 324.0637; found: 347.0541 [M + Na]^+^.

1,5-bis(4-(methylthio)phenyl)penta-1,4-diyn-3-one (**5**)

To a solution of Compound **4** (0.972 g, 3 mmol) in anhydrous CH_2_Cl_2_ (30 mL), MnO_2_ (1.043 g, 12 mmol) was added slowly at room temperature. Upon completion of the reaction, CH_2_Cl_2_ was removed in vacuum. The residue was extracted with water and dichloromethane, washed with saturated brine, dried over anhydrous sodium sulfate and concentrated under reduced pressure. The crude product was purified by flash chromatography to obtain compound **5** (yellow solid, 2.826 g, 94.2%) [26]. ^1^H NMR (400 MHz, CDCl_3_) δ 7.55 (d, *J* = 8.7 Hz, 4H), 7.23 (d, *J* = 8.5 Hz, 4H), 2.51 (s, 6H). ^13^C NMR (100 MHz, CDCl_3_) δ 160.77(1C), 144.21(2C), 133.77(4C), 125.44(4C), 115.22(2C), 92.18(2C), 90.21(2C), 14.92(2C).

### 3.2. STM-BJ Method

The single-molecule conductance was measured by scanning tunneling microscopy break junction (STM-BJ) technique using commercially available STM-BJ equipment provided by VR (Xiamen) Technology Co., Ltd. (Xiamen, China) in the 1,2,4-Trichlorobenzene (TCB) solution containing 0.1 mM of synthesized molecule [40]. The bias voltage applied between electrodes was 100 mV [33,41]. The retraction rate of electrodes was calibrated using the reported method in pure TCB solvent as a blank experiment (Figure S13) [42].

### 3.3. Computational Details

Geometry optimizations and orbital simulations of molecules were carried out by the Gaussian 16 software [31] based on the density functional theory (DFT) method employing the function of B3LYP with a 6-311+g (d,p) basis set and the vibrational frequencies were also calculated simultaneously with the same theoretical level to make sure that all the optimized configurations had the lowest energies. The DFT combined with non-equilibrium Green’s functions (NEGF) was used to simulate the transmission coefficient that corresponded to the transport properties.

The curves of the transmission coefficient were calculated by first-principles calculations using the Quantum Atomistix ToolKit software package, U-2022.12 version. The two electrodes, which were constructed from gold (111) surfaces, consisted of a unit cell of 5 × 5 with an extension region thickness of five layers to avoid strong interactions with electrodes. A pyramid-shaped gold tip was built to simulate the stable contact with anchors on both gold electrode sides. The above optimized molecular structures were then transformed between two electrodes to construct the single-molecule devices. For the initial configuration of the device, the distances between the sulfur atom and the top gold atom were controlled at ~2.5 Å. In the following optimized process, the coordinates of the gold tip atoms were frozen and molecular skeletons were kept rigid to save the computational cost and maintain the optimal conformation of the molecular structure. The exchange-correlation Generalized Gradient Approximation (GGA) with Perdew–Burke–Ernzerh (PBE) parameterization was employed in the simulations of the molecular device. In the simulation, the double-ζ basis set was adopted for gold atoms while the double-ζ polarization basis functions were applied to the other atoms. To meet the requirement of estimating the Au electrode system, the energy cutoff was set to 200 Ry. At zero bias voltage, the geometry optimization was optimized to meet the convergence criterion of 0.02 eV/A. The k point with a grid of (4 × 3 × 200) was set and a bias energy range from −3 eV to 3 eV was adopt in the transmission spectrum calculation.

## 4. Conclusions

We designed skipped-conjugated molecules using a hydroxyl group to build a hyperconjugation system. Single-molecule conductance measurements revealed an approximate two-fold conductance enhancement in the hyperconjugation system containing the ‘bridging’ hydroxyl group compared to the corresponding hydroxyl-free derivatives. The theoretical investigations demonstrated that the hydroxyl group in the skipped-conjugated structures (molecules **1** and **4**) set up a ‘bridge’ between the two conjugated fragments in LUMO, resulting in the visual similarity of the frontier orbitals to that of the fully conjugated structures (molecules **2** and **5**). Transmission studies further manifested that the connected orbitals of the two conjugated fragments in LUMO opened a through-space channel for electron transport and eventually enhanced the conductance. This study offers a rational strategy for designing hyperconjugation systems that integrate the benefits of conjugation and structural flexibility into a single molecule, thus providing insight into how the hyperconjugation systems facilitate electron transport in single-molecule devices.

## Data Availability

The raw data supporting the conclusions of this article will be made available by the authors on request.

## Supplementary Materials

The following supporting information can be downloaded at: https://www.mdpi.com/article/10.3390/molecules29112440/s1, Figure S1: ^1^H NMR Spectra of **1**; Figure S2: ^13^C NMR Spectra of **1**; Figure S3: ^1^H NMR Spectra of **2**; Figure S4: ^13^C NMR Spectra of **2**; Figure S5: ^1^H NMR Spectra of **3**; Figure S6: ^13^C NMR Spectra of **3**; Figure S7: ^1^H NMR Spectra of **4**; Figure S8: ^13^C NMR Spectra of **4**; Figure S9: ^1^H NMR Spectra of **5**; Figure S10: ^13^C NMR Spectra of **5**; Figure S11: ^1^H NMR Spectra of **6**; Figure S12: ^13^C NMR Spectra of **6**; Figure S13: One-dimensional conductance histogram, two-dimensional conductance histogram and the relative displacement distribution determined from 10^0.3^ *G*_0_ to 10^−6.0^ *G*_0_ using pure solvent for the calibration of the stretching rate of the electrodes pair; Figure S14: Two-dimensional conductance histograms and statistical junction lengths of molecules **1**–**6**; Figure S15: One-dimensional histograms of molecules **1**–**6** with the fitting curves of background (dashed brown lines), multimodal fitting (dashed grey lines) and the most probable conductance (colored peaks); Figure S16: Distributions of frontier molecular orbitals of HOMO and LUMO of molecules **1**–**6** in the previous report; Figure S17: Theoretically optimized molecular configurations and corresponding S-S distances of molecules **1**–**6**; Figure S18: Theoretical model used for transport calculations of single-molecule device; Figure S19: Transmission pathways of molecules **2** and **5**. Reference [29] are cited in the Supplementary Materials.

## References

[1] Jia C., Guo X. (2013). Molecule–electrode interfaces in molecular electronic devices. Chem. Soc. Rev..

[2] Su T.A., Neupane M., Steigerwald M.L., Venkataraman L., Nuckolls C. (2016). Chemical principles of single-molecule electronics. Nat. Rev. Mater..

[3] Li Q., Zhang Y., Lin J., Zou Y., Wang P., Qin Z., Wang Y., Li Y., Zhang Y., Gao C. (2023). Dibenzothiophene Sulfone-Based Ambipolar-Transporting Blue-Emissive Organic Semiconductors towards Simple-Structured Organic Light-Emitting Transistors. Angew. Chem. Int. Ed..

[4] Si W., Li J., Li G., Jia C., Guo X. (2024). Single-molecule non-volatile memories: An overview and future perspectives. J. Mater. Chem. C.

[5] Liu J., Huang X., Wang F., Hong W. (2018). Quantum Interference Effects in Charge Transport through Single-Molecule Junctions: Detection, Manipulation, and Application. Acc. Chem. Res..

[6] Vazquez H., Skouta R., Schneebeli S., Kamenetska M., Breslow R., Venkataraman L., Hybertsen M.S. (2012). Probing the conductance superposition law in single-molecule circuits with parallel paths. Nat. Nanotechnol..

[7] Cardamone D.M., Stafford C.A., Mazumdar S. (2006). Controlling Quantum Transport through a Single Molecule. Nano Lett..

[8] Chen H., Hou S., Wu Q., Jiang F., Zhou P., Zhang L., Jiao Y., Song B., Guo Q.-H., Chen X.-Y. (2021). Promotion and suppression of single-molecule conductance by quantum interference in macrocyclic circuits. Matter.

[9] Chen H., Zheng H., Hu C., Cai K., Jiao Y., Zhang L., Jiang F., Roy I., Qiu Y., Shen D. (2020). Giant Conductance Enhancement of Intramolecular Circuits through Interchannel Gating. Matter.

[10] Lo W.Y., Bi W., Li L., Jung I.H., Yu L. (2015). Edge-on Gating Effect in Molecular Wires. Nano Lett..

[11] Li L., Louie S., Evans A.M., Meirzadeh E., Nuckolls C., Venkataraman L. (2023). Topological Radical Pairs Produce Ultrahigh Conductance in Long Molecular Wires. J. Am. Chem. Soc..

[12] Li L., Low J.Z., Wilhelm J., Liao G., Gunasekaran S., Prindle C.R., Starr R.L., Golze D., Nuckolls C., Steigerwald M.L. (2022). Highly conducting single-molecule topological insulators based on mono- and di-radical cations. Nat. Chem..

[13] Zhen S., Mao J.-C., Chen L., Ding S., Luo W., Zhou X.-S., Qin A., Zhao Z., Tang B.Z. (2018). Remarkable Multichannel Conductance of Novel Single-Molecule Wires Built on Through-Space Conjugated Hexaphenylbenzene. Nano Lett..

[14] Zhen S., Shen P., Li J., Zhao Z., Tang B.Z. (2021). Giant single-molecule conductance enhancement achieved by strengthening through-space conjugation with thienyls. Cell Rep. Phys. Sci..

[15] Shen P., Huang M., Qian J., Li J., Ding S., Zhou X.S., Xu B., Zhao Z., Tang B.Z. (2020). Achieving Efficient Multichannel Conductance in Through-Space Conjugated Single-Molecule Parallel Circuits. Angew. Chem. Int. Ed..

[16] Alabugin I.V., Kuhn L., Krivoshchapov N.V., Mehaffy P., Medvedev M.G. (2021). Anomeric effect, hyperconjugation and electrostatics: Lessons from complexity in a classic stereoelectronic phenomenon. Chem. Soc. Rev..

[17] Vedernikova I., Salahub D., Proynov E. (2003). DFT study of hyperconjugation effects on the charge distribution in pyrogallol. J. Mol. Struct. Theochem.

[18] Tostes J.G.R., Seidl P.R., Soto M.M., Carneiro J.W.d.M., Lie S.K., Taft C.A., Brown W., Lester W.A. (1995). Ab initio studies of hyperconjugation effects on charge distribution in tetracyclododecane alcohols. Chem. Phys. Lett..

[19] Alabugin I.V., dos Passos Gomes G., Abdo M.A. (2018). Hyperconjugation. Wires Comput. Mol. Sci..

[20] Paddon-Row M.N. (2002). Some aspects of orbital interactions through bonds: Physical and chemical consequences. Acc. Chem. Res..

[21] Tang C., Jiang X.-L., Chen S., Hong W., Li J., Xia H. (2023). Stereoelectronic Modulation of a Single-Molecule Junction through a Tunable Metal–Carbon dπ–pπ Hyperconjugation. J. Am. Chem. Soc..

[22] Su T.A., Li H., Steigerwald M.L., Venkataraman L., Nuckolls C. (2015). Stereoelectronic switching in single-molecule junctions. Nat. Chem..

[23] Salthouse R.J., Hurtado-Gallego J., Grace I.M., Davidson R., Alshammari O., Agraït N., Lambert C.J., Bryce M.R. (2023). Electronic Conductance and Thermopower of Cross-Conjugated and Skipped-Conjugated Molecules in Single-Molecule Junctions. J. Phys. Chem. C.

[24] Cui J., Wong Y.L., Zeller M., Hunter A.D., Xu Z. (2014). Pd Uptake and H_2_S Sensing by an Amphoteric Metal–Organic Framework with a Soft Core and Rigid Side Arms. Angew. Chem. Int. Ed..

[25] Zhou B., Wu Q., Dong Z., Xu J., Yang Z. (2019). Rhodium-Catalyzed 1,1-Hydroacylation of Thioacyl Carbenes with Alkynyl Aldehydes and Subsequent Cyclization. Org. Lett..

[26] Praveen Rao P.N., Chen Q.-H., Knaus E.E. (2006). Synthesis and Structure–Activity Relationship Studies of 1,3-Diarylprop-2-yn-1-ones: Dual Inhibitors of Cyclooxygenases and Lipoxygenases. J. Med. Chem..

[27] Verma A.K., Fatima K., Dudi R.K., Tabassum M., Iqbal H., Kumar Y., Luqman S., Mondhe D.M., Chanda D., Khan F. (2020). Antiproliferative activity of diarylnaphthylpyrrolidine derivative dual target inhibition. Eur. J. Med. Chem..

[28] Hu Z.G., Liu J., Zeng P.L., Dong Z.B. (2019). Synthesis of α, α′-bis(Substituted Benzylidene)Ketones Catalysed by a SOCl_2_/EtOH reagent. J. Chem. Res..

[29] Guo M.M., Jiang Y.X., Wang J.Y., Chen Z.N., Hou S.M., Zhang Q.C. (2024). Effectively Enhancing the Conductance of Asymmetric Molecular Wires by Aligning the Energy Level and Symmetrizing the Coupling. Langmuir.

[30] Lin J., Lv Y., Song K., Song X., Zang H., Du P., Zang Y., Zhu D. (2023). Cleavage of non-polar C(sp2)–C(sp2) bonds in cycloparaphenylenes via electric field-catalyzed electrophilic aromatic substitution. Nat. Commun..

[31] Frisch M.J., Trucks G.W., Schlegel H.B., Scuseria G.E., Robb M.A., Cheeseman J.R., Scalmani G., Barone V., Petersson G.A., Nakatsuji H. (2016). Gaussian 16 Rev. B.01.

[32] Pan H., Wang Y., Li J., Li S., Hou S. (2022). Understanding Quantum Interference in Molecular Devices Based on Molecular Conductance Orbitals. J. Phys. Chem. C.

[33] Yan S.-S., Chen L.-C., Wang J.-Y., Duan P., Pan Z.-Y., Qu K., Hong W., Chen Z.-N., Zhang Q.-C. (2023). Exploring a Linear Combination Feature for Predicting the Conductance of Parallel Molecular Circuits. Nano Lett..

[34] Lambert C.J., Liu S.X. (2018). A Magic Ratio Rule for Beginners: A Chemist’s Guide to Quantum Interference in Molecules. Chemistry.

[35] Lambert C.J. (2015). Basic concepts of quantum interference and electron transport in single-molecule electronics. Chem. Soc. Rev..

[36] Chen L.C., Zheng J., Liu J., Gong X.T., Chen Z.Z., Guo R.X., Huang X., Zhang Y.P., Zhang L., Li R. (2020). Nonadditive Transport in Multi-Channel Single-Molecule Circuits. Small.

[37] Tostes J.R., Seidl P.R., Taft C.A., Lie S.K., Carneiro J.W.d.M., Brown W., Lester W.A. (1996). Carbon–carbon and carbon–hydrogen hyperconjugation in neutral alcohols. J. Mol. Struct. Theochem.

[38] Nokhbeh S.R., Gholizadeh M., Salimi A., Sparkes H.A. (2020). Synthesis, crystal structure, Hirshfeld surface analysis, DFT calculations and characterization of 1,3-propanediylbis(triphenylphosphonium) monotribromide as brominating agent of double bonds and phenolic rings. J. Mol. Struct..

[39] Schultz K.P., Spivey D.W., Loya E.K., Kellon J.E., Taylor L.M., McConville M.R. (2016). Photochemical locking and unlocking of an acyl nitroso dienophile in the Diels–Alder reaction. Tetrahedron Lett..

[40] Xu B., Tao N.J. (2003). Measurement of Single-Molecule Resistance by Repeated Formation of Molecular Junctions. Science.

[41] Pan Z., Dong G., Shang C., Li R., Gao T., Lin L., Duan H., Li X., Bai J., Lai Y. (2023). XMe-Xiamen Molecular Electronics Code: An Intelligent and Open-Source Data Analysis Tool for Single-Molecule Conductance Measurements. Chin. J. Chem..

[42] Hong W., Manrique D.Z., Moreno-García P., Gulcur M., Mishchenko A., Lambert C.J., Bryce M.R., Wandlowski T. (2012). Single Molecular Conductance of Tolanes: Experimental and Theoretical Study on the Junction Evolution Dependent on the Anchoring Group. J. Am. Chem. Soc..

