# Supplementary material for: Hydroxyl Group as the ‘Bridge’ to Enhance the Single-Molecule Conductance by Hyperconjugation
Source: Molecules. 2024 May 22;29(11):2440. doi: 10.3390/molecules29112440 (PMC11173964; doi:10.3390/molecules29112440)
Supplement: Supplementary file 1 [file molecules-29-02440-s001.zip › molecules-3000293-supplementary.pdf]

Supplementary Material for:

Hydroxyl Group as the 'Bridge' to Enhance the Single-Molecule Conductance by Hyperconjugation

Xin Lv [1,2,3], Chang Li [1,4], Meng-Meng Guo [1], Wenjing Hong [5], Li-Chuan Chen [5,*], Qian-Chong Zhang [1,3,6,*] and Zhong-Ning Chen [1,3,6]

[1] Key Laboratory of Structural Chemistry, Fujian Institute of Research on the Structure of Matter, Chinese Academy of Sciences, Fuzhou 350002, China; lvxin@fjirsm.ac.cn (X.L.); lichang@fjirsm.ac.cn (C.L.); mengmengguo@fjirsm.ac.cn (M.-M.G.); czn@fjirsm.ac.cn (Z.-N.C.)
[2] College of Chemistry and Materials Science, Fujian Normal University, Fuzhou 350007, China
[3] Fujian College, University of Chinese Academy of Sciences, Fuzhou 350002, China
[4] University of Chinese Academy of Sciences, Beijing 100049, China
[5] State Key Laboratory of Physical Chemistry of Solid Surfaces, College of Chemistry and Chemical Engineering, Xiamen University, Xiamen 361005, China; whong@xmu.edu.cn
[6] Fujian Science & Technology Innovation Laboratory for Optoelectronic Information of China, Fuzhou 350108, China
* Correspondence: chenlichuan@xmu.edu.cn (L.-C.C.); zhangqianchong@fjirsm.ac.cn (Q.-C.Z.)

1.NMR Spectra

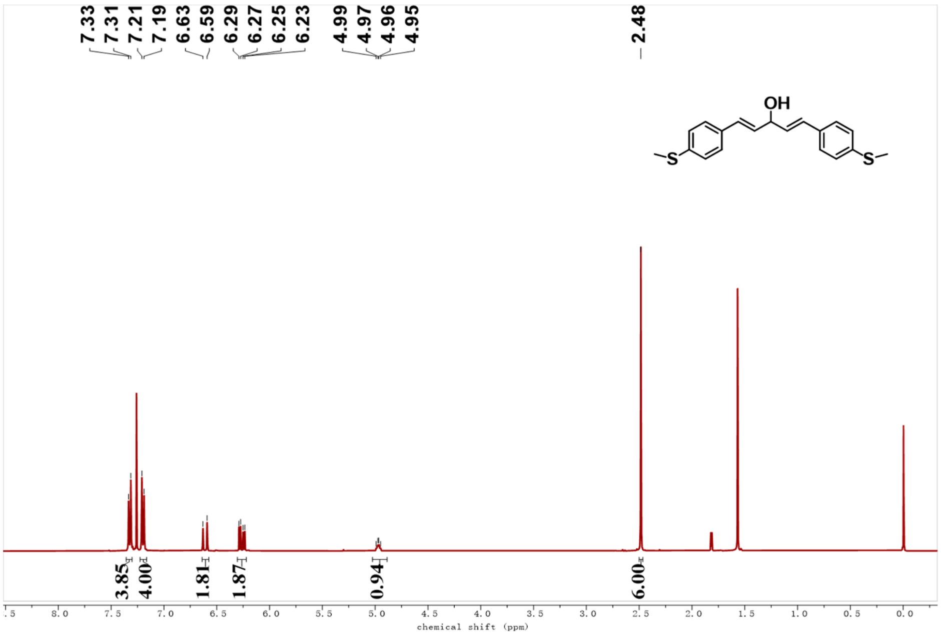

Figure S1 ^{1}H NMR Spectra of **1**.

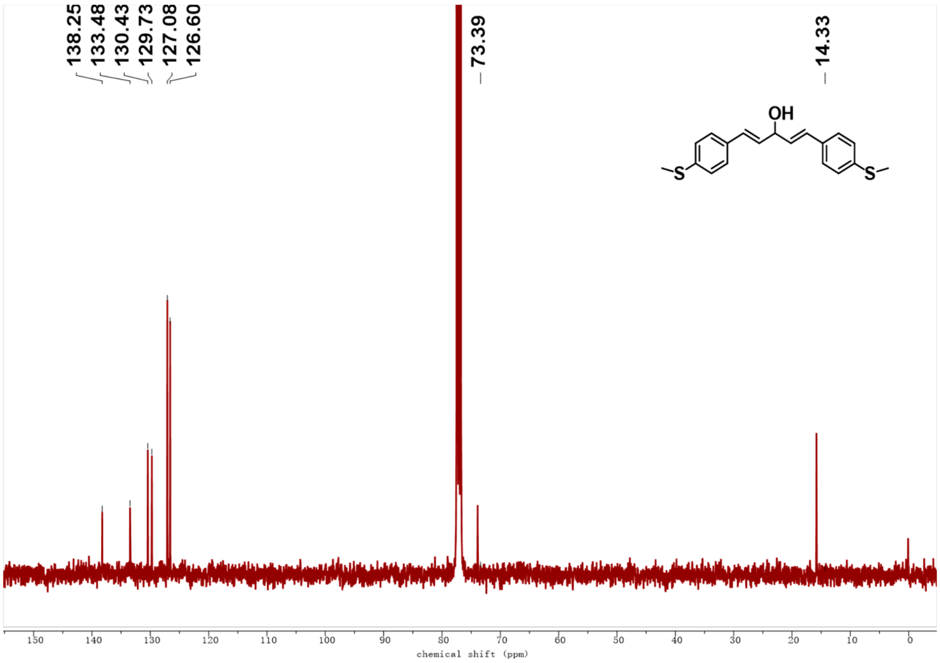

Figure S2 ^{13}C NMR Spectra of **1**.

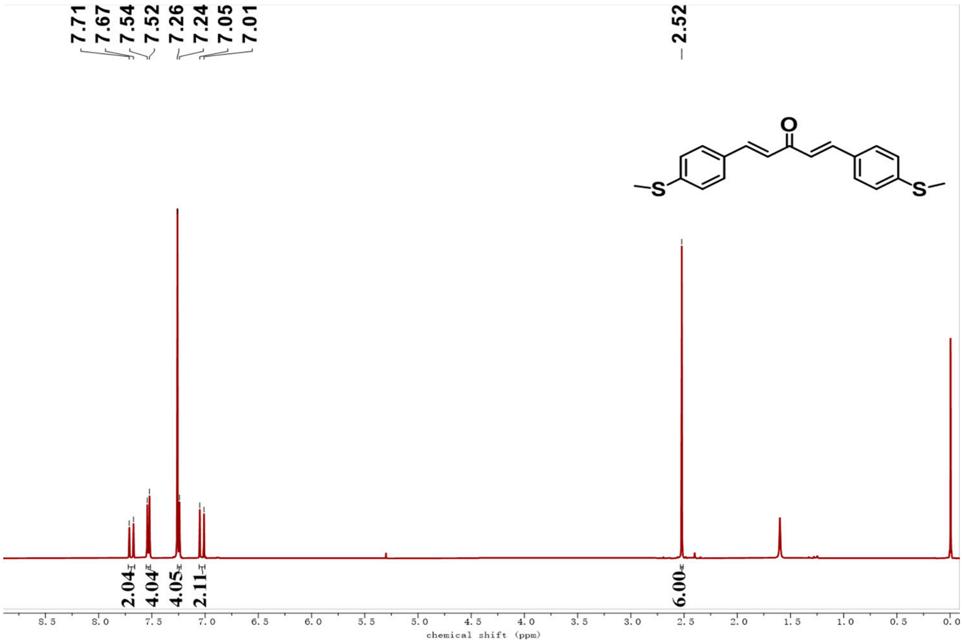

Figure S3 ^{1}H NMR Spectra of **2**.

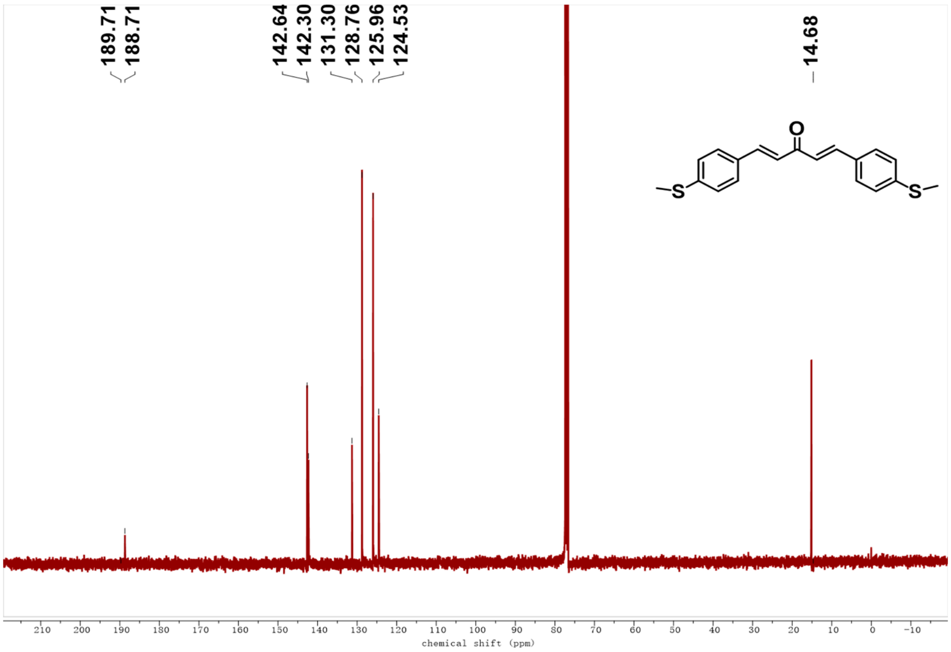

Figure S4 ^{13}C NMR Spectra of **2**.

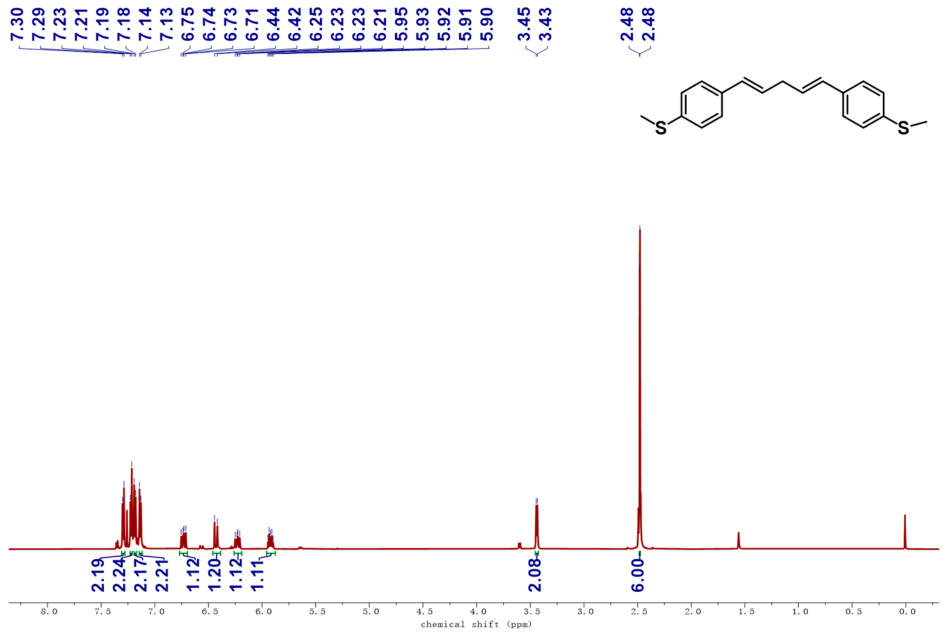

Figure S5 ^{1}H NMR Spectra of **3**.

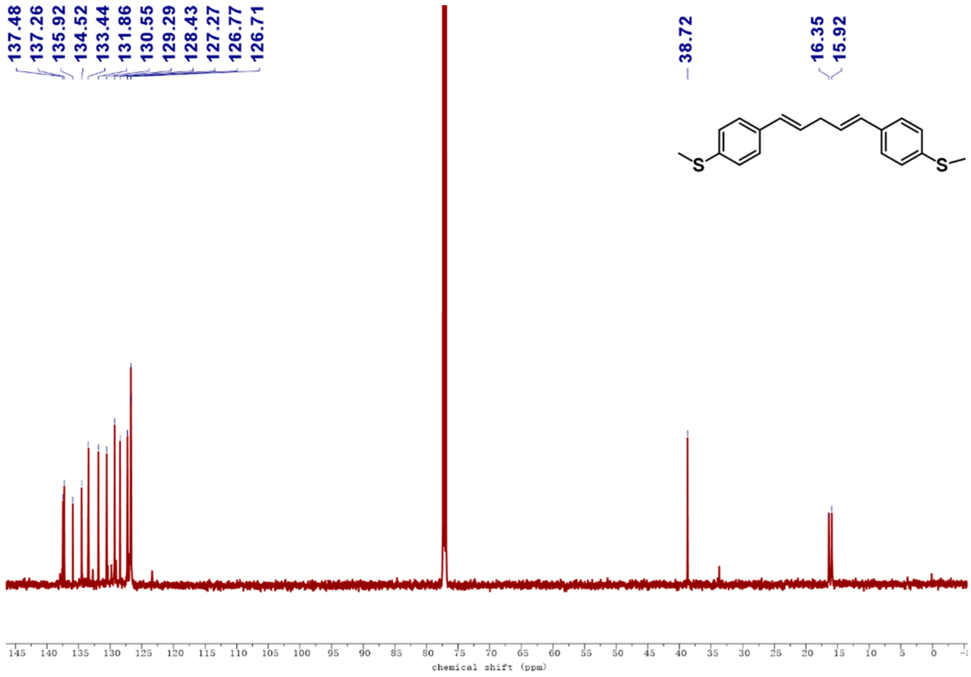

Figure S6 ^{13}C NMR Spectra of **3**.

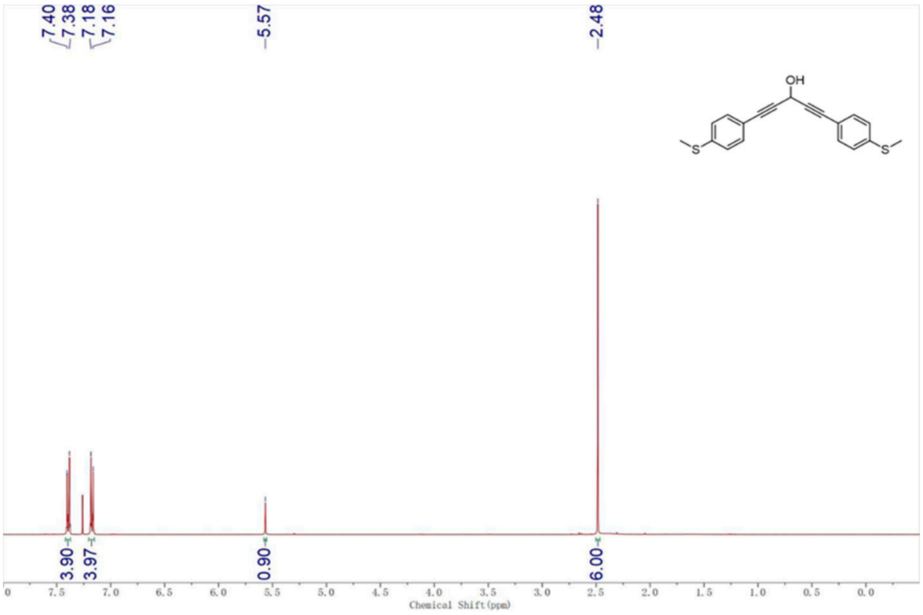

Figure S7 ^{1}H NMR Spectra of **4**.

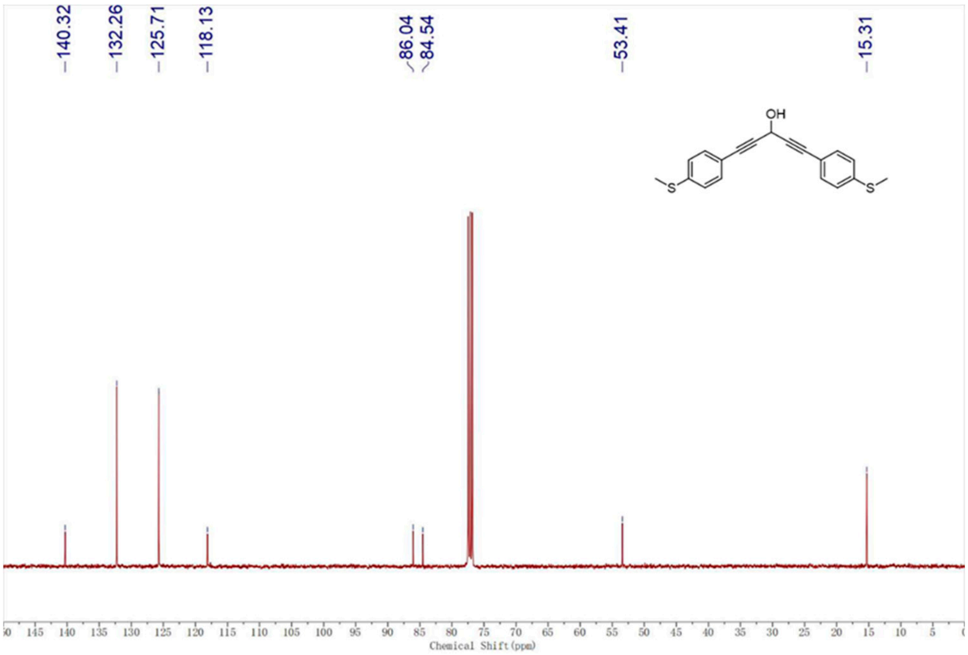

Figure S8 ^{13}C NMR Spectra of **4**.

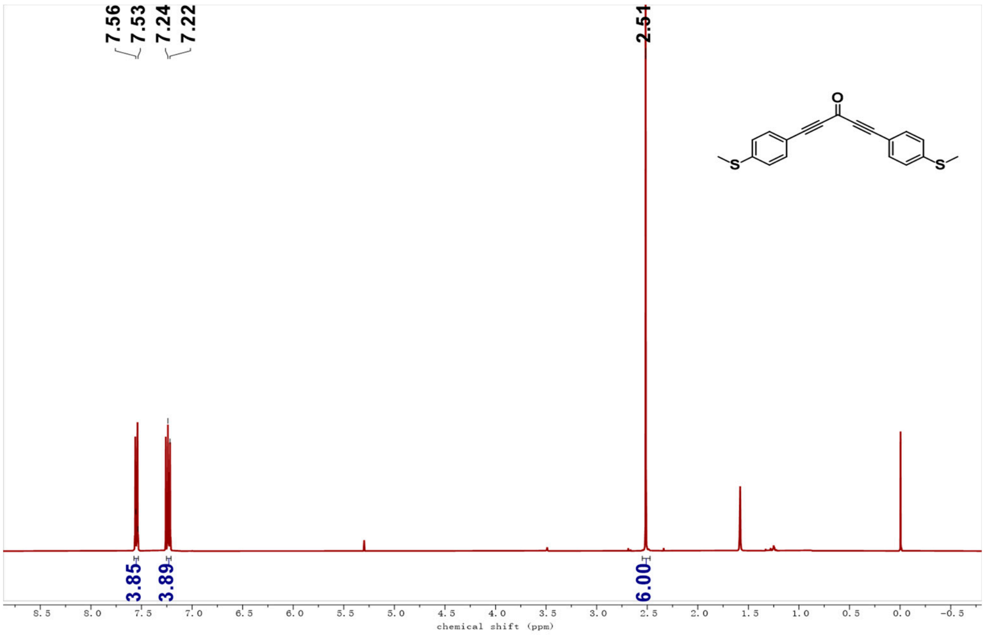

Figure S9 ^{1}H NMR Spectra of **5**.

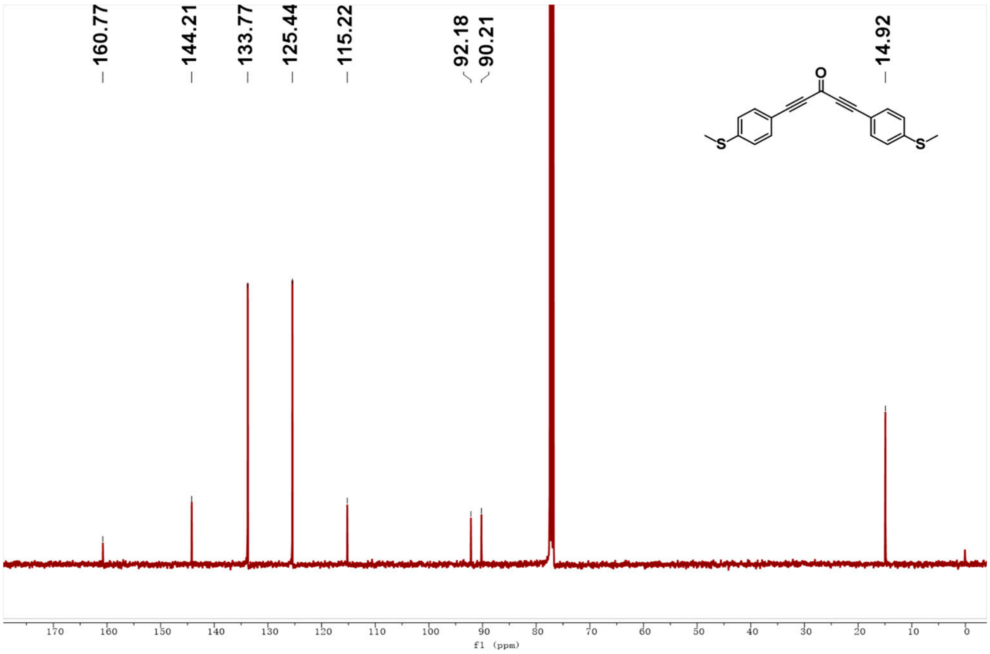

Figure S10 ^{13}C NMR Spectra of **5**.

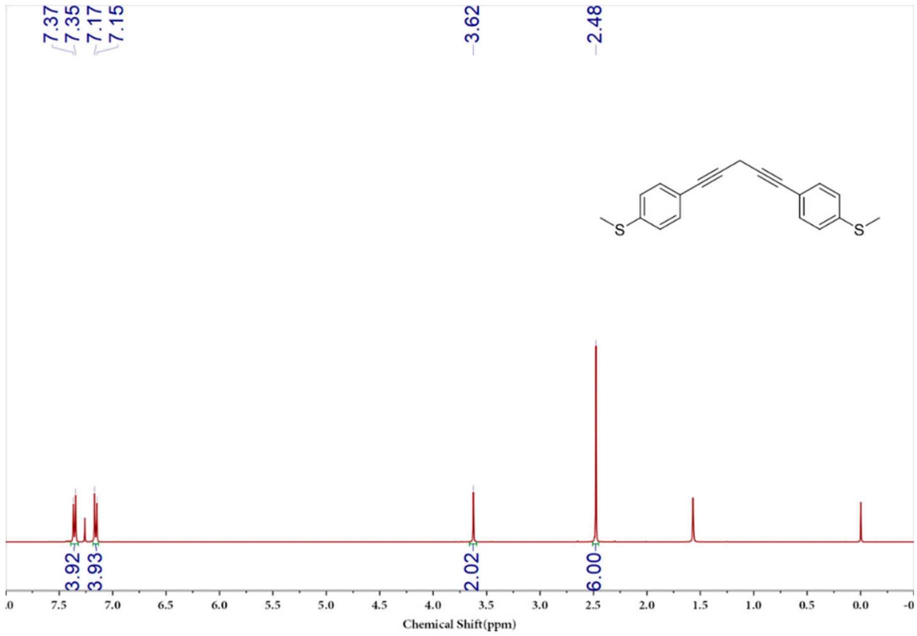

Figure S11 ¹H NMR Spectra of **6**.

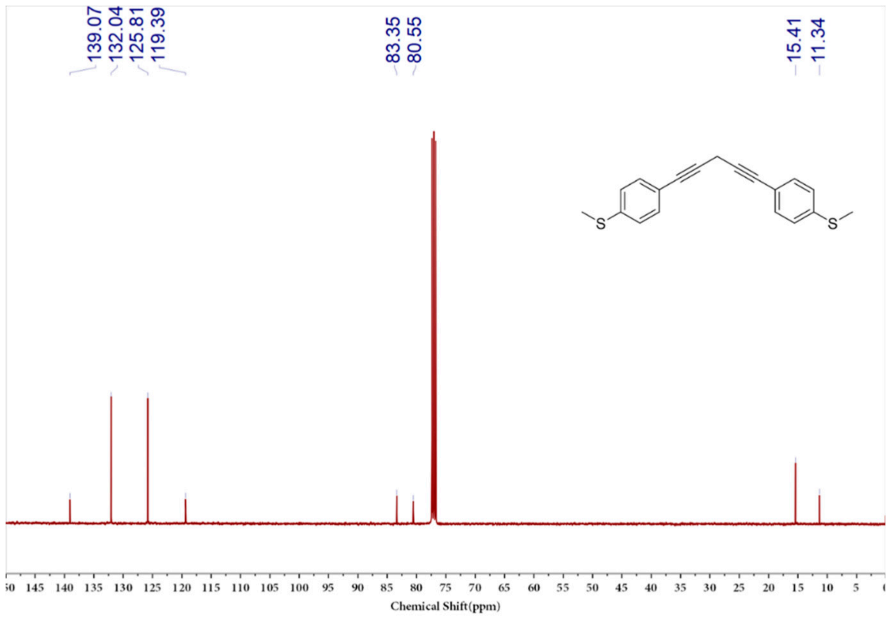

Figure S12 ¹³C NMR Spectra of **6**.

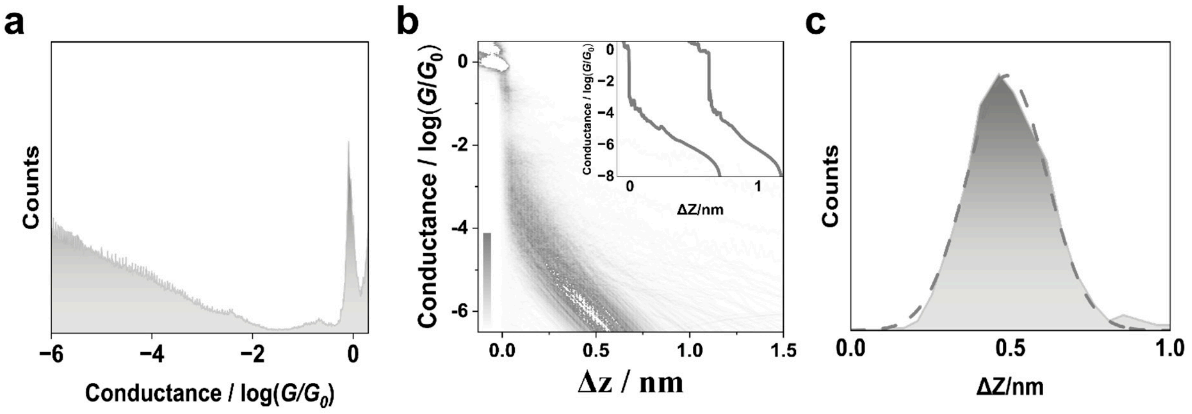

Figure S13 One-dimension conductance histogram, two-dimension conductance histogram and the relative displacement distribution determined from $10^{0.3}$ G_0 to $10^{-6.0}$ G_0 using pure solvent for the calibration of the stretching rate of the electrodes pair.

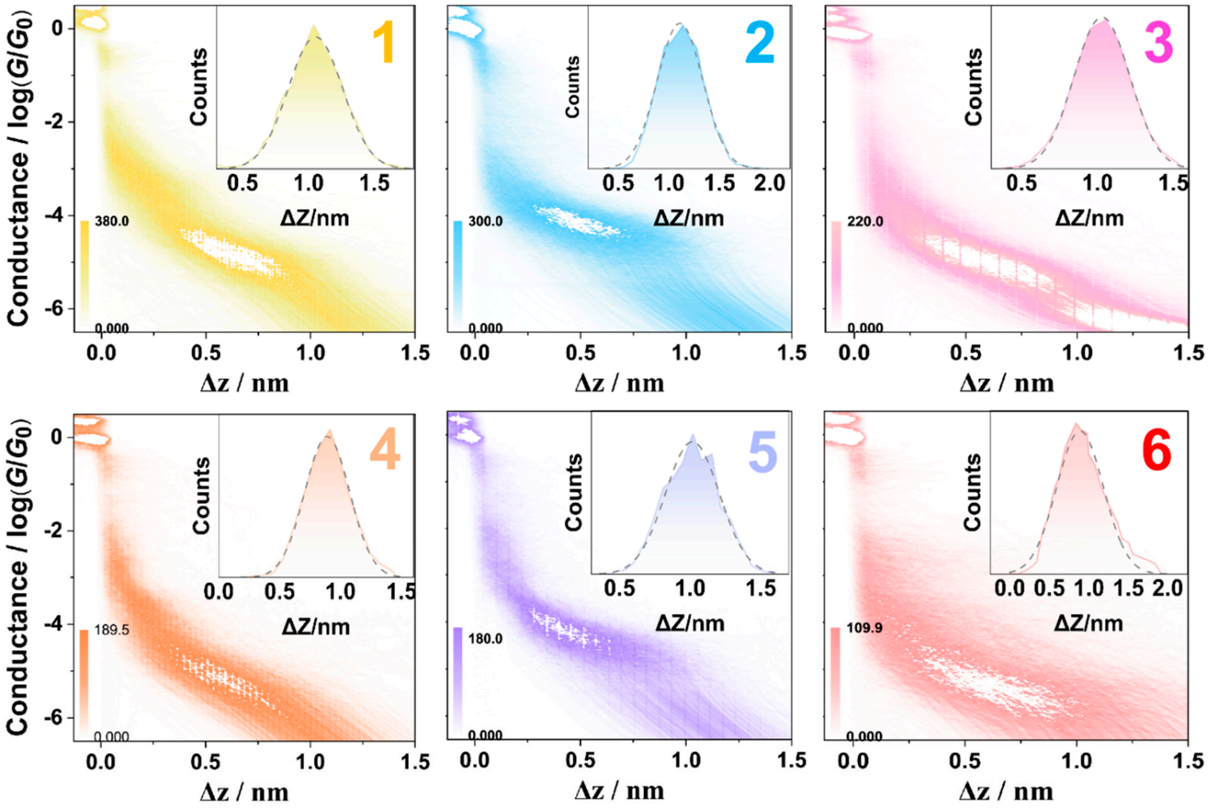

Figure S14 Two-dimensional conductance histograms and statistical junction lengths of molecules **1-6**.

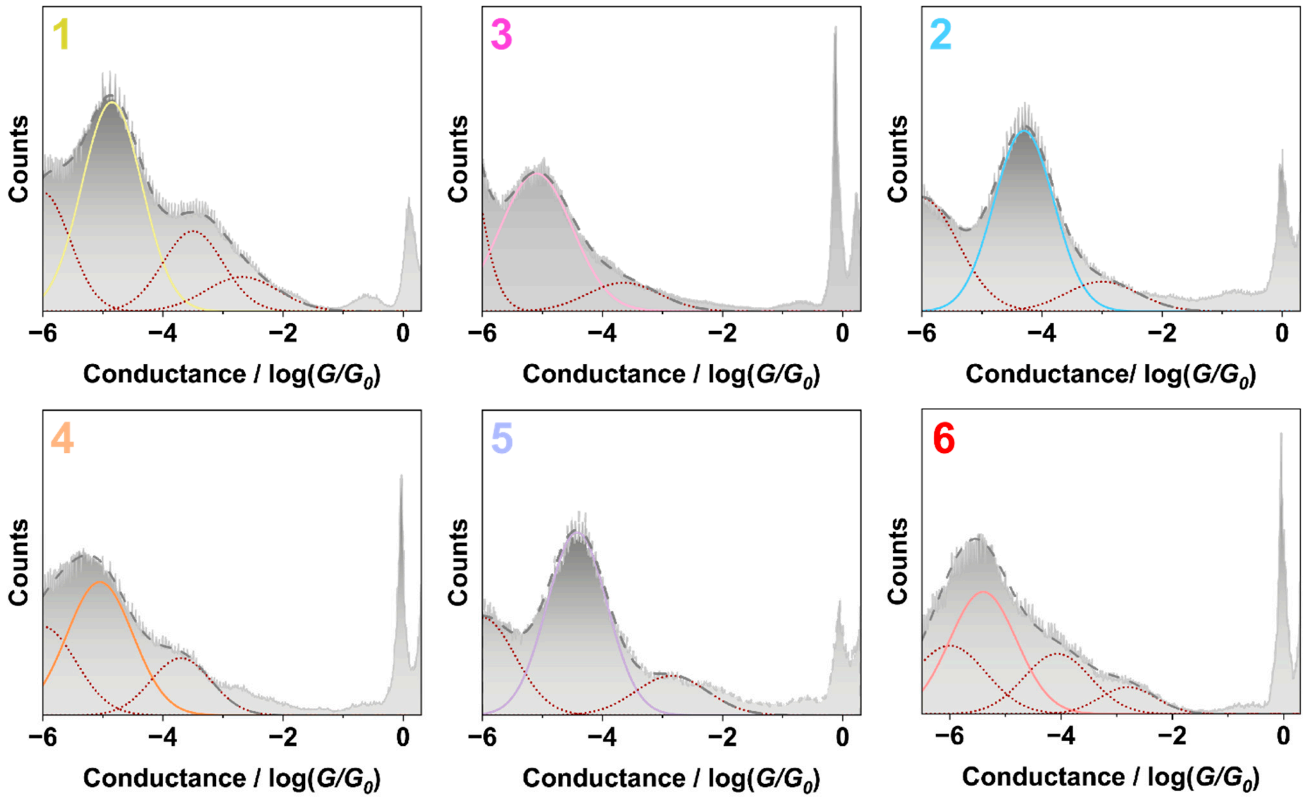

Figure S15 One-dimensional histograms of molecules **1-6** with the fitting curves of background (dashed brown lines), multimodal fitting (dashed grey lines) and the most probable conductance (colored peaks).

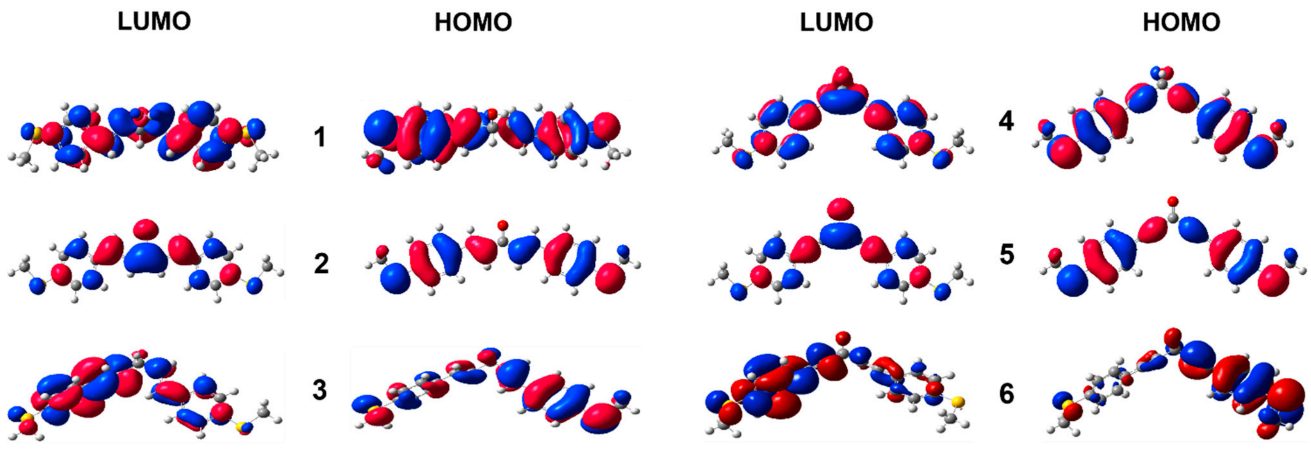

Figure S16 Distributions of frontier molecular orbitals of HOMO and LUMO of molecules **1-6** in the previous report [1]. Reproduced with permission from

ref. [1]. 2024 American Chemical Society.

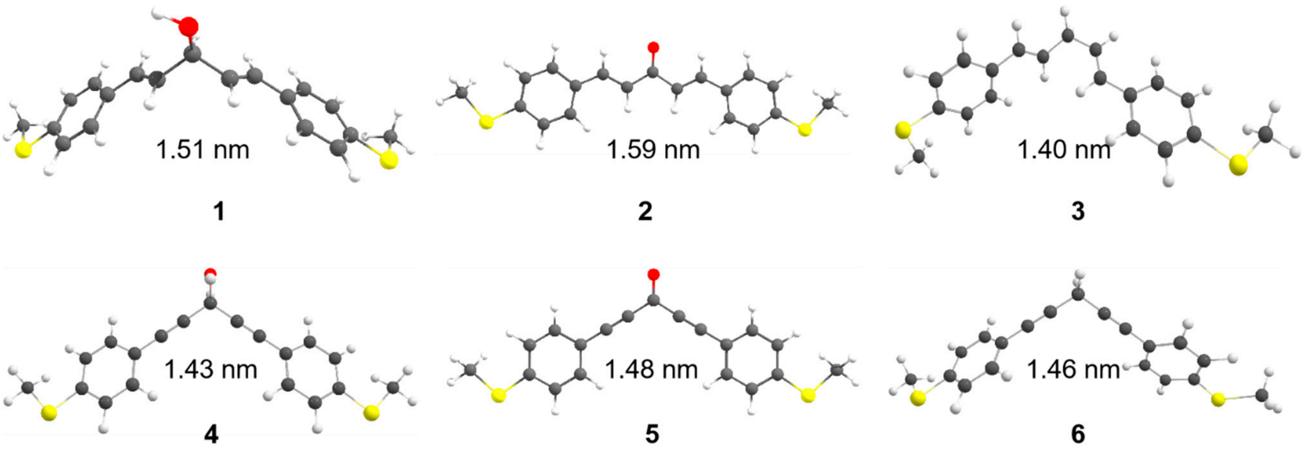

Figure S17 Theoretically optimized molecular configurations and corresponding S-S distances of molecules **1-6**.

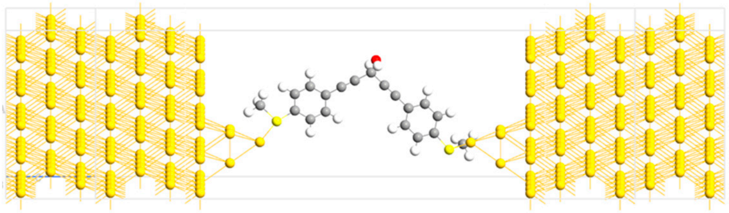

Figure S18 Theoretical model used for transport calculations of single-molecule device.

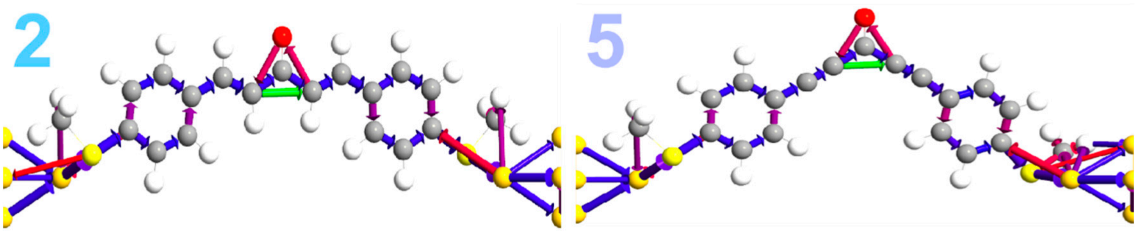

Figure S19 Transmission pathways of molecules **2** and **5**

Notes and references

1. Guo, M. M.; Jiang, Y. X.; Wang, J. Y.; Chen, Z. N.; Hou, S. M.; Zhang, Q. C., Effectively Enhancing the Conductance of Asymmetric Molecular Wires by Aligning the Energy Level and Symmetrizing the Coupling. *Langmuir* **2024,** 40, (7), 3759-3765. http://dx.doi.org/10.1021/acs.langmuir.3c03530
